# Design and Fabrication of Biosensor for a Specific Microbe by Silicon-Based Interference Color System

**DOI:** 10.3390/mi15060741

**Published:** 2024-05-31

**Authors:** Muthusamy Sivakumar, Sangami Ervanan, Susithra Lakshmanan, Sathya Venkatesan, Takatoshi Kinoshita, Duraikkannu Shanthana Lakshmi, Alagarsamy Santhana Krishna Kumar

**Affiliations:** 1 Department of Materials Science and Engineering, Nagoya Institute of Technology, Gokiso-cho, Showa-ku, Nagoya 4668555, Japan; kinoshita.takatoshi@nitech.ac.jp; 2 St Catharine’s College, University of Cambridge, Trumpington St, Cambridge CB2 1RL, UK; sangamirayan@gmail.com; 3 Cambridge Centre for Advanced Research and Education in Singapore, 1 Create Way, CREATE, Singapore 138602, Singapore; susithra02@gmail.com; 4 Department of Chemistry, AMET Deemed to be University, Chennai 603112, India; sathyass028@gmail.com; 5 RSK Environment Ltd., 18 Frogmore Road, Hempstead HP3 9RT, UK; slakshmi@rsk.co.uk; 6 Department of Chemistry, National Sun Yat-sen University, No. 70, Lienhai Road, Gushan District, Kaohsiung 80424, Taiwan

**Keywords:** iridescent color, silicon, biosensor, RNA aptamer, bacteria, XPS

## Abstract

In this paper, one of the great challenges faced by silicon-based biosensors is resolved using a biomaterial multilayer. Tiny biomolecules are deposited on silicon substrates, producing devices that have the ability to act as iridescent color sensors. The color is formed by a coating of uniform microstructures through the interference of light. The system exploits a flat, RNA-aptamer-coated silicon-based surface to which captured microbes are covalently attached. Silicon surfaces are encompassed with the layer-by-layer deposition of biomolecules, as characterized by atomic force microscopy and X-ray photoelectron spectroscopy. Furthermore, the results demonstrate an application of an RNA aptamer chip for sensing a specific bacterium. Interestingly, the detection limit for the microbe was observed to be 2 × 10^6^ CFUmL^−1^ by visually observed color changes, which were confirmed further using UV-Vis reflectance spectrophotometry. In this report, a flexible method has been developed for the detection of the pathogen *Sphingobium yanoikuyae*, which is found in non-beverage alcohols. The optimized system is capable of detecting the specific target microbe. The simple concept of these iridescent color changes is mainly derived from the increase in thickness of the nano-ordered layers.

## 1. Introduction

In recent years, silicon substrate has come to be considered a potential platform for the development of biosensors. Biosensors have emerged as a highly promising platform for the rapid diagnosis of microbes in foodstuffs. The development of different strategies to immobilize biomaterials onto silicon substrates has resulted in the improvement of biosensors. In this study, we present a novel type of display system in the form of an optical sensor platform to solve the problem of microbe diagnosis encountered by the food industry. The majority of sensors are based on labeling the probe with fluorescence-modified [1] or radioactive-labeled targets [2]. Indeed, our laboratory fabricated a chameleon-type iridescent color system using several nano-ordered layers of polypeptide on silicon substrate [3]. However, colorimetric detection systems have a fundamental problem detecting specific molecular interactions, such as antibodies or nucleic acids. At the same time, the advantage of the optical platform is that the unaided eye can detect changes in the iridescent color due to several factors such as the nano-ordered film thickness, the number of layers, and the angle of the incident light [3,4,5,6,7]. The chip presented herein is developed especially for the detection of a specific pathogen, *Sphingobium yanoikuyae* [8,9], which alters the taste of non-beverage alcohols. Recently, we have reported a *fujike* RNA aptamer chip fabricated on a polypeptide monolayer that is attached to a silicon surface, and the chip finds application in the field of biosensing [10]. However, it does not show a significant iridescent color except for a small shift in the wavelength due to the very thin monolayer. To address this problem, in this study, we pursue two key objectives; one is increasing the thickness of the coating biomaterials through a multilayer immobilization technique that facilitates an improvement in significant iridescent color changes; and the second is to improve the binding activity of the biomaterial by choosing a different RNA aptamer, namely, pSy-14, which consists of 102 nucleotides derived from *pGEM-3Z* Vectors by the SELEX process, which has increased binding activity against the specific target microbe compared to *fujike* RNA. Recently, many oligonucleotide sensors have been developed on silicon or gold surfaces [11,12] to target proteins and microbes. Similarly, a silicon-chip-based biosensor with biotin-coated nitrocellulose membrane was reported and its surface was attached with poly- and monoclonal antibodies for the detection of *salmonella* in poultry [13]. Surface-modified Si was also used to bind biomolecules, especially those pertaining to specific cell types [14]. Sensors based on acoustic waves [15], electrochemical methods [16], and the electronic nose [17] are well known for the detection of bacteria. Since those sensors have limited diagnostic ability, they require instrumentation for signal detection [18]. We have worked for several years on silicon-based iridescent color chips through the coating of various numbers of peptide layers [3,5,6]. In this study, we have investigated the application of different numbers of nano-ordered layers of different biomaterials through a step-by-step surface-modification strategy, which provides considerable flexibility in the formation of an iridescent color response for visual sensor applications. Herein, the direct attachment of organic molecules to Si/SiO_2_ is accomplished by silanization reaction [19]. The multilayered chip has also been stabilized by biotin–avidin interaction in the core of the layers. This current work represents a novel type of colorimetric sensor that has certain merits in sensing a pathogen. Furthermore, this report covers the objective of fabrication of an aptamer-based multilayered chip and the detection of a target microbe by changes in the iridescent color response.

## 2. Materials and Methods

A silicon wafer (100) chip (diameter of 7 mm and thickness of 1 mm) was procured from The Nilaco Co., Tokyo, Japan. 3-aminopropylmethyldiethoxysilane (APMES) was purchased from Gelest Inc. (Morrisville, PA, USA). Biotinamidohexanoic acid 3-sulfo-N-hydroxysuccinimide ester sodium salt (Biotin-AC_5_-sulfo-Osu) was purchased from Dojindo, Japan. Avidin (from Egg white) and biotinylated polythymine30 (BiotindT30, Biotin-(3′-T30mer)) were obtained from Sigma-Aldrich Co., USA and Funakoshi Co., Ltd., Japan, respectively. Sy14 RNA aptamer (72dA30mer, 72nt polyAdenine, and (Sy aptamer-5′ A30mer 3′) and *Sphingobium yanoikuyae* bacteria were kindly provided by Prof. Kikuchi, Toyohashi University of Technology and Prof. Hiraishi, Toyohashi University of Technology, Japan, respectively.

### 2.1. Surface Preparation

**Scheme 1:**
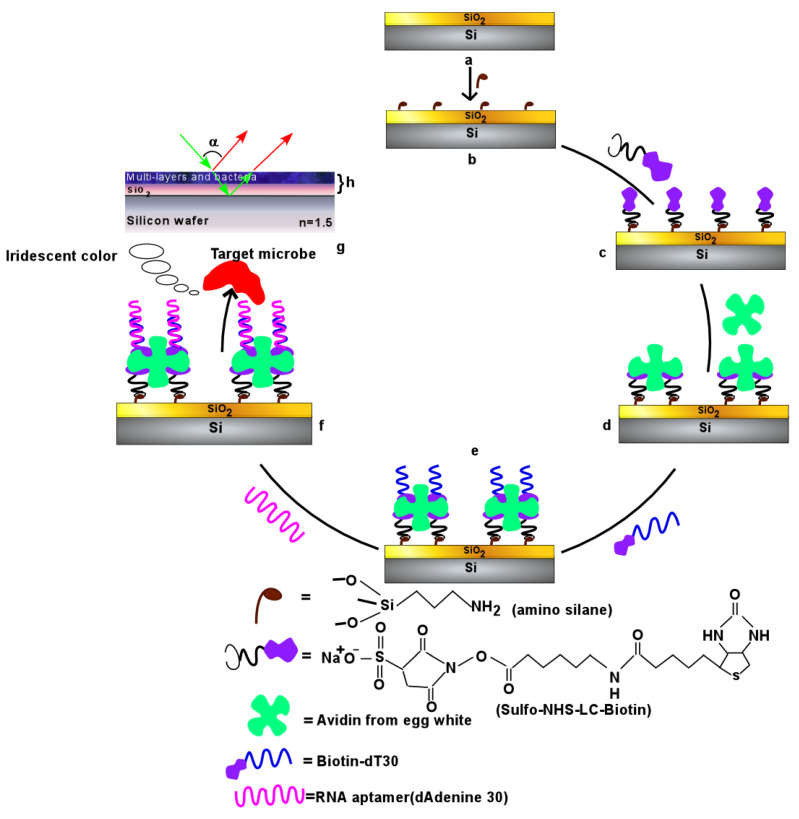
Fabrication of silicon-based iridescent color system for biosensor application. (**a**) SiO_2_-oxidized, (**b**) aminated, (**c**) biotin, (**d**) avidin, (**e**) BiotindT30, (**f**) RNA aptamer, and (**g**) formation of iridescent color while binding with bacteria.

The silicon wafer was sintered at high temperatures (~1080 °C for 4.5 h) to obtain the required color substrate (Si/SiO_2_). Furthermore, Si/SiO_2_ plates were subjected to chemical oxidation as reported earlier by Davis et al. [20], which allowed subsequent modifications. The detailed procedure of the layer-by-layer deposition is as follows:

The dried iridescent-color silicon chip (Scheme 1a) was placed in a toluene solution of 3-aminopropylmethydiethoxy silane (0.1 volume %) for 120 min at 25 °C with continuous N_2_ purge. This silanized plate (Scheme 1b) was fixated at 110 °C for 20 min in air and then rinsed with toluene, toluene/methanol (1:1), and methanol for a few minutes with sonication. All immobilization studies were conducted in phosphate buffer pH: 7.4 at 25 °C for 24 h unless otherwise specified. 

Biotin-Sulfo-Osu-AC_5_ (1 × 10^−2^ mL^−1^) was attached to the surface of the silica through an amino-functionalized derivative (Scheme 1c), producing a biotin-terminated surface (B).

The biotinylated chip was then immersed into an avidin (1 × 10^−5^ mL^−1^) solution. The avidin-modified chip (Scheme 1d) was rinsed with phosphate buffer solution to remove unbound avidin.

In order to immobilize RNA aptamer, the avidin plate was immersed into a biotin dT30 oligonucleotide solution (2 ng/μL) and then the surface was dried. Next, the resultant chip (Scheme 1e) was rinsed with phosphate buffer solution to remove unbound material.

Finally, RNA aptamer was diluted as 2 ng/μL in Tris-EDTA buffer (TE) (10 mM) and immobilized onto the BiotindT30 surface for 24 h at 25 °C and then rinsed with TE buffer to remove excess aptamer from the surface (Scheme 1f).

Finally, the RNA aptamer chip was immersed in bacterial solutions of different concentrations from 2 × 10^2^ to 2 × 10^9^ CFUmL^−1^, individually, at 25 °C for 6 h. Afterward, the immersed chips were rinsed with binding buffer to remove unbound bacteria followed by MilliQ water, and finally dried at 37 °C in an incubator for 1 h. The different layers of biomaterials were coated on Si/SiO_2_ substrate, which is schematically described in Scheme 1. First, surface hydroxyl groups of the silicon surface were silanized with 3-aminopropylmethyldiethoxysilane. This –NH_2_-modified surface was allowed to react with a Biotin-Sulfo-Osu-AC_5,_ which resulted in the formation of a biotinylated surface. Subsequently, avidin was attached to this biotinylated substrate. Furthermore, biotinylated polythymine was modified on the avidin surface. A final layer of single-strand RNA aptamer probe was immobilized onto the respective base pair of the polythymine30 surface. The complementary sequence of adenine (30mer) in RNA aptamer is well stabilized through hybridization onto the biotinylated thymine (30mer). The remaining 72nt of RNA aptamer has the capacity for biorecognition and can virtually recognize the target microorganism, *Sphingobium yanoikuyae*, with high affinity and specificity.

### 2.2. Surface Characterization

#### 2.2.1. X-ray Photoelectron Spectroscopy (XPS)

XPS measurements were done using a Surface Science Instruments X-probe SSX-100 XPS spectrometer equipped with a monochromatic Al Kα X-ray source and concentric hemispherical electron energy analyzer. The take-off angle was 45° under high vacuum. Spectra of the layer-by-layer modified silica surfaces were recorded with 50 eV pass energy and a spot size of 600 μm × 600 μm. Three spots are measured per sample chip.

#### 2.2.2. Atomic Force Microscopy (AFM)

A Nanoscope IV (Digital Instruments Inc.) atomic force microscope in tapping mode was used to obtain images of the modified silicon surfaces at ambient temperature. Images were recorded in the constant-force mode using sharpened silicon nitride tips mounted on cantilevers with a nominal force constant.

#### 2.2.3. UV-Vis Reflectance Spectrophotometry

The aptamer chip was analyzed using UV-Vis reflectance spectrophotometry and a Light Interference type Environmental Sensor (LIFES-5501, Moritex Corporation, Japan) from wavelength 400 nm to 800 nm at an incident angle of 10°. The bacteria-bound chips were also analyzed using the LIFES-5501 to confirm the color change.

## 3. Results and Discussion

### 3.1. Surface Analysis by XPS

**Figure 1:**
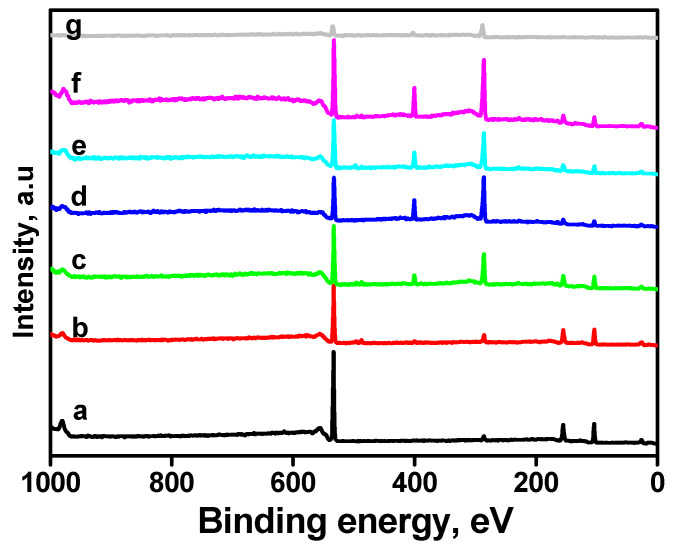
XPS survey spectra of different layers, deposited (layer-by-layer) on silicon surface. (a) SiO_2_-oxidized, (b) aminated, (c) biotin, (d) avidin, (e) BiotindT30, (f) RNA aptamer, and (g) bacteria-bound surfaces.

XPS was used to monitor the layer-by-layer modification of biomaterials as it can provide information on chemical structure, atomic concentration, and surface contamination. XPS analysis of the surface revealed the covalent nature of the surface modification. The binding energies of C, N, and O on the modified surfaces were compared with that of bare silica substrate and are shown in Figure 1. Each element has a specific binding energy and yields a characteristic set of peaks in the photoelectron spectrum, which reveals its presence in the layer. The binding energy of C (1s), O (1s), and N (1s) are approximately 286 eV, 532 eV, and 401 eV, respectively. P2 (3/2) and S (2p) are not clearly observed in the survey spectrum due to their lower concentration. However, the independent spectra show the presence of P2 (3/2) and S (2p). The positions of these peaks coincide well with the values given in the literature [21]. The N (1s) peak at around 401 eV was found in the XPS spectra of all multilayered depositions but was absent in the spectrum of the bare substrate of silica. This finding indicated a building up of nitrogen-containing moieties on the silicon surface. The Si (2p) core-level spectra also display a prime peak at 103.4 eV, due to highly oxidized Silica [22]. In addition, the bacteria-bound surface was characterized and showed peaks for C (1s), O (1s), and N (1s), although the peak intensities were relatively low, which shows that the nitrogen element is less available in the bacteria. As expected, no signals were observed at Si (2s) (150.3 eV) and Si (2p) (99.3 eV), as in the case of the multilayered chips, due to the shallow depth penetration of soft X-rays. These results demonstrate that there is an extra thickness of bacteria, which is confirmed by later experiments, discussed below. These results also demonstrate that the binding between the aptamer and bacteria is not physical.

**Figure 2:**
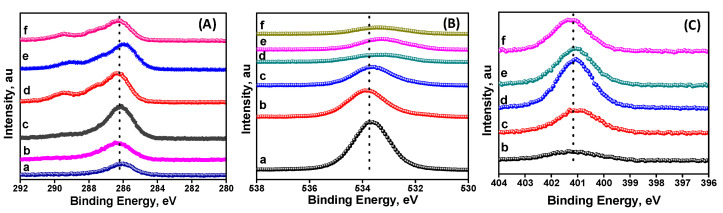
XPS spectra of different surfaces of modified silicon substrate with different elements (**A**): C (1s), (**B**): O (1s), and (**C**): N (1s). (a) SiO_2_-oxidized, (b) aminated, (c) biotin (d), avidin (e) BiotindT30, and (f) RNA aptamer.

**Figure 3:**
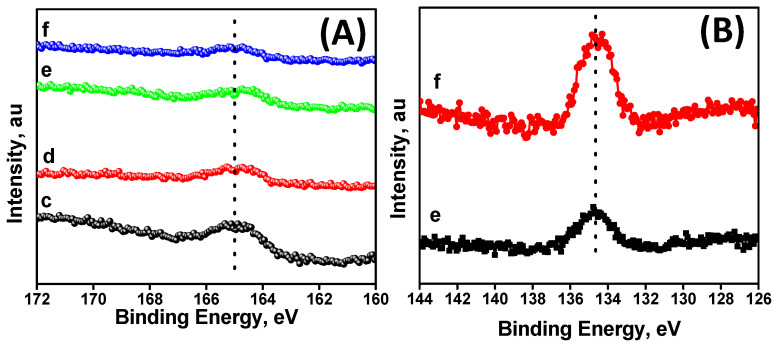
XPS spectra of different surfaces of modified silicon substrate with different elements (**A**) S (2p3) and (**B**) P (2p3/2). (c) biotin, (d) avidin, (e) BiotindT30, and (f) RNA aptamer.

As Figure 2A shows, the bare silicon surface (a) shows a very small hump corresponding to the C (1s) peak, the intensity of which increases with each modification. The peak position also shows significant changes in the chemical nature of the species from the bare Si/SiO_2_ to the RNA-aptamer-modified surface. The small humps corresponding to the C (1s) peak observed for the bare Si wafer/silica substrate could be attributed to atmospheric impurities; however, the peaks of some oxidized forms, i.e., C=O, C–O bonding, are also observed in the range 286.3 to 287.2 eV [23]. The appearance of a peak at 285.9 eV for C–N bonding is due to the aminated surface, which confirms the formation of an amine surface on the silica surface [21]. Three types of carbon species (hydrocarbon, 284.8 eV, ether carbon, 286.6 eV, and amido carbon, 284 eV) are observed in different layers (Figure 3A). Furthermore, two other carbon peaks are observed at slightly higher binding energies (amido carbon, 289.5, ether carbon, 287.5, and ketone carbon, 286.3) in the avidin, BiotindT30, and aptamer layers, respectively [23]. The peak at 287.9 eV is characteristic of O=C–N environments [21] and the peak at 289.0 eV is typical of –COOH bonding. Figure 2B shows the changes in the binding energy of the O (1s) from 532.6 eV to 535.0 eV. The peak corresponds to the contribution of O (1s) to the bare Si wafer at 532.6 eV and to the SiO_2_ substrate at 533.8 eV (Figure 2B(a)). The binding-energy peaks confirm the different amounts of oxygen content after annealing and chemical oxidation of the Si wafer. The O (1s) signal of the silanized surface is at 533.69, and the small shift observed is probably due to the high coverage of the silica surface due to silanization. Furthermore, the O (1s) peak of the biotin (O=C–NH) surface is expected at 533.6 eV and overlaps with the siloxane signals. The carboxyl groups (–COOH) from avidin (which includes several amino acids) appeared over a wide range of binding energies between 535.5 and 532.0 eV. The increase in the binding energy confirmed the strong noncovalent binding of avidin onto the biotin surface. Furthermore, the BiotindT30 and aptamer surfaces show peaks at 533.6 and 533.4 eV, respectively, which again suggests that there is no physical bonding in the case of biotin–avidin interactions. Furthermore, an increase in binding energy was observed until the RNA aptamer surface. 

As Figure 2C shows, the N (1s) band is observed near 400 eV for the aminosilylated surface (Scheme 1b), which suggests a C–NH_2_ bonding (free amino groups). This is in good agreement with the previous report by Xiao [24] for aminopropyltriethoxy silane. As expected, there is an increase in N content as we move from an amine to the aptamer surface. Furthermore, the consecutive peaks at binding energies of 401 eV to 401.5 eV are attributed to the amide that exists in the Biotin-AC_5_-Sulfo-Osu and avidin layers, respectively. This clearly distinguishes it from free amino groups (of the first layer). The increase in the binding energy of the avidin layer is due to the strong bonding ability between biotin and avidin. The avidin layer was covered on the biotinylated chip as the next layer (Scheme 1d). Furthermore, the biotinylated polythymine30 (BiotindT30) surface shows a peak at around 401 eV, and the reduction in the binding energy indicates that it could be attributed to the biotin or polythymine. The peak in the last layer of RNA aptamer is also observed in the same binding-energy region as that of N (1s) but appears at 401.38 eV. The small broad peak is due to the final layer of aptamer in which imine nitrogen is also included. However, the higher binding energy (i.e., above 401 eV) of the layers from Scheme 1b–d is due to nitrogen involved in oxidized environments (C–N–O bonding). 

Figure 3A shows the high-resolution XPS S (2p^3^) spectrum for the Biotin-Sulfo-Osu-AC_5_, avidin, BiotindT30, and aptamer multilayers. Sulfur signals could be seen on all the layers except for the first silanized layer. This provides direct evidence for the presence of sulfur in the other layers of Biotin-Sulfo-Osu-AC_5_ and the second layer of biotin (Scheme 1b) consisting of C–S–CH and C–S–H bonding. The broad peaks near 164 eV for the biotin and avidin layers are due to the presence of the C–S–C and C–S–H functional groups, respectively. This binding-energy peak shows obvious agreement with the reported value of sulfur chemically bound to a gold surface (i.e., the formation of a gold–thiolate bond) [25,26]. Moreover, the binding energy is shifted to 164.5 and 163.6 eV for BiotindT30 and RNA aptamer, respectively. However, no sulfur is available on the top two layers. Figure 3B shows that phosphorus is present in the top two layers (Scheme 1d,e) out of several layers. The nucleotides available in the top two layers (BiotindT30 and RNA aptamer) have phosphate groups. P (2p^3^) peaks of biotinylated polythymine 30 (BiotindT30) appear at 134.6 eV. This result proves that the terminal group of biotin in polythymine 30 is tethered to the previous layer of avidin by non-covalent bonding. The bond formation between biotin and avidin is very rapid and, once formed, is unaffected by wide extremes of pH, temperature, organic solvents, and other denaturing agents. Furthermore, RNA aptamer (sy14 aptamer) was immobilized onto the BiotindT30 surface and has a slightly wider peak between 134 and 136 eV. This indicates that the top layer was base-paired and that hydrogen bonds were present between the bases (adenine and thymine). The quantitative information (atomic concentrations) derived from the XPS intensities are summarized in Table 1. The values depend on the measured volume and the chemical components. After annealing and chemical oxidation, no contaminants except for carbon were found on the surfaces. However, no traces of sulfur and nitrogen exist on those chemically oxidized surfaces. Indeed, the average atomic concentration reflects the sequential reactions and even the presence of N element in lower concentrations after silanization. It is well known that exposed surfaces of Si become spontaneously covered with an approximately 200 nm layer of oxide, mostly as SiO_2_, during high-temperature calcination. The increase in the quantity of oxygen element shows that a suitable iridescent color chip was obtained, as shown in Table 1. 

Basically, the content of oxygen in the silica substrate increases due to spontaneous oxidation, as evident from Table 1, and then decreases again due to its coverage by aminosilane. The oxygen content gradually decreases until the avidin layer then increases slightly in the BiotindT30 layer due to the availability of oxygen in biotin and thymine and finally slight changes in the RNA aptamer layer. This also proved that the molecules added through layer-by-layer modification contain adequate amounts of oxygen element. The first, aminated layer showed a carbon concentration of approximately 25%, which is comparatively less than that of the biotinylated surface (~45%) (Table 1), and is attributed to the long alkyl chains located in Biotin-Sulfo-Osu-AC_5_. Furthermore, the carbon content increased gradually in the following avidin, BiotindT30, and aptamer layers. The relative amount of carbon element increased from the silica to avidin layers since the avidin monomer contains 128 amino acid residues [27]. The content of carbon decreased slightly is moving to the BiotindT30 (Scheme 1e) layer due to the penetration of soft X-rays to only a short depth, and at this stage, the XPS process of photoionization might be carried out as part of the avidin layer and remaining BiotindT30. Finally, with the inclusion of the RNA aptamer layer, the carbon content increased slightly. The N (1s), in the lowest amount, is present in all types of multilayer deposition, but not in the bare substrate, and the amount of N (1s) slightly increased in the aminated layer and then increased in a step-wise manner until the aptamer surface. The bacteria-bound surface was also observed and showed a small decrease in N (1s) due to the limited amount of nitrogen element available in the bacterial structure. Since the XPS experiment was conducted in fixed take-off angle (45°) mode and with partial depth analysis, Si also steadily decreased from the bare substrate to the aptamer layer, which is clearly evident from Table 1. The layers were deposited one by one and the multilayer thickness increased concurrently. 

### 3.2. Aptamer Chip Binding with Microbe

**Figure 4:**
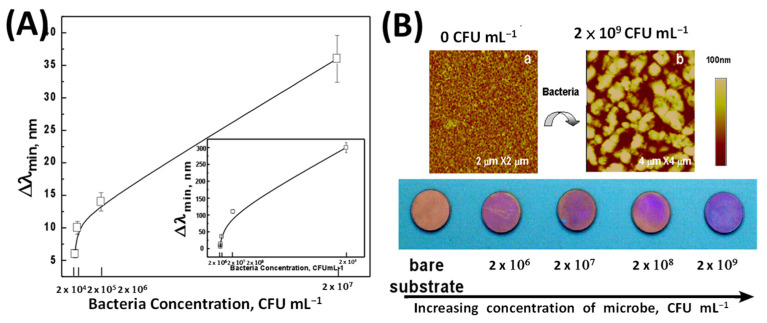
(**A**) The binding isotherms between RNA aptamer probe and bacteria on the fabricated iridescent color response chips. Inset figure for higher concentrations of bacteria. (**B**) AFM images of the aptamer chip before and after binding with microbes and real images of color chips after immersing into different concentrations of bacteria [(a) at initial concentration and (b) final concentration], respectively.

The resulting aptamer chip was next tested with the bacteria *Sphingobium yanoikuyae* at different concentrations in a 2 mL solution in phosphate buffer. The receptor of aptamer bound through its ligand to the bacteria due to the presence of the specific-binding envelope in the respective *Sphingobium*. The terminal of the single-strand RNA sequence was modified at the C-7 position in adenine. Concurrently, to measure the selectivity of the aptamer chip, we used a different bacterium, *E. coli*. The receptor, RNA aptamer, was not functionally modified to bind *E. coli*, thus it could not bind this or any other pathogen. Binding studies were then conducted with different concentrations (from 1 × 10^2^ to 1 × 10^9^ CFUmL^−1^) of *Sphingobium yanoikuyae* microbes and analyzed by UV-Vis. The changes in ∆λ_min_ occurred mainly from 8 to 302 nm for different concentrations of bacteria between 2 × 10^5^ and 2 × 10^9^ CFUmL^−1^, respectively, and are shown in Table 2. The data were plotted as ∆λ_min_ versus bacteria concentration and are shown as a binding isotherm in Figure 4A.

However, more binding activity may be observed when the concentration of microbes is increased. Moreover, the membrane of the microbe was targeted by the respective stem-loop secondary structure of the aptamer sequence [28,29]. Here, the ligands of the aptamer bind with high affinity to a specific site of the microbe envelope. The inset in Figure 4A shows the binding isotherm for higher concentrations of bacteria. Indeed, the lower concentrations (2 × 10^2^~2 × 10^5^ CFUmL^−1^) of microbes do not show a significant ∆λ_min_ compared with higher concentrations. Interestingly, the color of the chip gradually changes from gold to blue [30], which is due to the interference of colors with respect to the increase in the concentration of the bacteria from 2 × 10^5^ CFUmL^−1^ to 2 × 10^9^ CFUmL^−1^ (inset of Figure 4A). A significant change in reflectivity was also observed in the spectrum due to good reactivity between RNA aptamer and the target bacteria. The bio-device surfaces are designed such that a minimum thickness increase causes a color change, which can be clearly seen by the human eye. The entire topology of the resultant chips was analyzed using AFM and more bacterial coverage (for the highest concentration of 2 × 10^9^ CFUmL^−1^) was observed, as shown in Figure 4B. As a result, the bacterial colony was very evident. The bacterium is rod-shaped (bacillus) and has a diameter of 100–200 nm [10]. The beautiful iridescent color of the oxide/organic layer is formed due to the periodic arrangement of oxide/organic materials (Scheme 1g). This arises through the interference and reflection of scattering light, which is referred to as an iridescence. The iridescent color response chip changes from gold to blue due to the binding of higher concentrations of bacteria, which is shown in Figure 4B (real chip images). The data were put into the interference of light [31] equations. The modified multilayered thickness (*h*) was calculated using the following equations.

(1) $$\lambda_{\max}=\frac{2h}{m}\sqrt{n^{2}-\sin^{2}\alpha }$$

(2) $$\lambda_{\begin{array}{l} \min \end{array}}=\frac{4h}{2m-1}\sqrt{n^{2}-\sin^{2}\alpha }$$

where α is the incident angle (15° in the experiment), *m* is an arbitrary integer number, (*m* = 1, 2…), and *n* is the refractive index of the thin layer and is ~1.5. The observed average value is *h* = 444 nm and this was substituted into Equations (1) and (2), yielding an average *λ*_max_ = 650 nm, and, when *m* = 2, *λ*_min_ = 521 nm, and when *m* = 3, *λ*_max_ = 444 nm. Thus, when *m* = 3, *h* is similar to the observed values of *h* in UV-Vis reflectance spectra of the highest microbe concentration (2 × 10^9^ CFUmL^−1^)-bound chip. This may represent the entire thickness of the external layer on the silicon wafer. In fact, we can calculate the whole thickness of the external layer including the layers of oxide (SiO_2_), organic layers, and microbe. Strong shifts in optical properties were similarly observed when increasing the thickness by the addition of nanolayers [3], chemical vapor absorption [6,32], and an aqueous dispersion of microgel [33]. 

The average thickness of the oxide layer is approximately 240 nm and the remaining layers consist of multiple layers of biomaterials including the microbe. The average value of bacterial thickness was shown to be 30 nm, which was estimated from the cross-sectional analysis of the AFM image [10]. Nevertheless, the thickness increased enormously due to the aggregation of more specific microbes (numerous folds) (Figure 4B), and the average total thickness calculated for all layers was approximately 420 nm including the oxide layer. This will create a color change, which is attributed to the presence of a surface consisting of a multilayer nanostructure [5]. The oxide layer also plays a prominent role in the light interference that leads to a color response. A few other approaches to the visual detection of biomolecules have also been described other than microbe detection, such as optically coated silicon for nucleic acids [30], oxidized aluminum surfaces for proteins [34], and silicone nitride for the detection of PCR products [35]. Here, the color change is caused by the presence of a target molecule, which results in thickness changes in the order of a few hundred Å on the surface. The interaction causes iridescent color changes, which depend on the properties of the silicon surface modified by coating it with several layers of biomaterials. This type of aptamer-based visual sensor can also be applied for the selective sensing of metal ions [36] and other food contaminants.

## 4. Conclusions

A Si/SiO^2^ substrate was modified successfully using a layer-by-layer deposition process. Furthermore, biotin–avidin binding was stabilized by the multilayered structure as well by the increase in the thickness of the surface film, which promoted iridescent color changes. The RNA aptamer chips were tested with Sphingobium bacteria and verified by UV-Vis reflectance spectrophotometry in addition to visual observation by the naked eye. The fabricated iridescent color response chip was demonstrated to function as a biologically hybrid silicon probe that is sensitive to a specific microbe and its detection process is suitable for use in visual sensors. Nevertheless, although the sensitivity of the fabricated aptamer chip was good at higher concentrations of bacteria, currently, a thorough understanding of the factors inducing the binding characteristics, e.g., the binding constant between aptamer and bacteria, is lacking and research has to be carried out in this direction in the future. A key intention of this report is to accomplish optical detection by the detection of color variations, which is one of the suitable means of sensing a specific target without the use of any data transfer and extra display units and is inexpensive. Visual sensors based on iridescent color response are appealing and might have potential applications in areas such as food contamination and combatting bioterrorism.

## Figures and Tables

**Table 1:** Atomic concentrations of different elements determined by XPS.

Different Surfaces	Si (%)	O (%)	C (%)	N (%)
Si	35.3 (2.7%)	47.5 (2.9%)	7.15 (3.4%)	0 (0%)
SiO_2_	25.7 (3.2%)	62.7 (3.3%)	11.5 (3.7%)	0 (0%)
APMES	22.0 (2.5%)	48.1 (2.8%)	24.7 (3.2%)	3.2 (1.0%)
Biotin	18.1 (3.6%)	33.7 (3.9%)	43.1 (4.2%)	4.8 (3.7%)
Avidin	8.5 (2.5%)	21.7 (2.0%)	62.9 (3.1%)	10.8 (1.9%)
Biotin dT	8.4 (3.9%)	27.9 (3.8%)	53.7 (2.9%)	10.9 (1.7%)
RNA aptamer	8.4 (1.0%)	27.0 (1.8%)	57.8 (2.1%)	11.0 (2.0%)
Bacteria	0 (0%)	22.2 (1.7%)	66.9 (2.4%)	10.9 (1.5%)

**Table 2:** Wavelength (∆λ_min_) changes in UV-Vis reflectance spectra after immersing the RNA aptamer chip into the bacteria, *Sphingobium yanoikuyae* solution at different concentrations.

Concentration of Bacteria, CFUmL^−1^	2 × 10^2^	2 × 10^3^	2 × 10^5^	2 × 10^6^	2 × 10^7^	2 × 10^8^	2 × 10^9^
∆λ_min_, nm	0	8	10	14	36	110	302

## Data Availability

The data that support the findings of this study are available from the corresponding author upon reasonable request.
